# The role of polygenic indices in inequality of opportunity

**DOI:** 10.1093/pnasnexus/pgaf140

**Published:** 2025-05-05

**Authors:** Michael Grätz, Sonia Petrini

**Affiliations:** Swiss Centre of Expertise in Life Course Research LIVES, University of Lausanne, Lausanne 1015, Switzerland; Swedish Institute for Social Research SOFI, Stockholm University, Stockholm 10691, Sweden; Swiss Centre of Expertise in Life Course Research LIVES, University of Lausanne, Lausanne 1015, Switzerland

**Keywords:** education, equality of opportunity, income, occupation, polygenic indices

## Abstract

Equality of opportunity is a principle of social justice, although there are different conceptions of it. We distinguish between liberal and radical (in)equality of opportunity. Both conceptions consider unfair inequalities in life outcomes that result from ascribed characteristics such as social origin, migration background, and sex. However, they differ in that liberal inequality of opportunity considers it fair when natural talents affect life outcomes. Conversely, radical inequality of opportunity places natural talents in the category of morally arbitrary factors that do not provide a fair basis for inequalities in life outcomes. We use polygenic indices (PGIs) to better disentangle the role of natural talents from the roles of ascribed characteristics and individual choices. We compare using PGIs to using measures of skills observed later in life. We apply this approach to two survey datasets, (i) the Wisconsin Longitudinal Study and (ii) the German Socio-Economic Panel Study. According to our results, radical inequality of opportunity is considerably larger than liberal inequality of opportunity, especially for education. Measuring natural talents using PGIs leads to very similar conclusions as using skills measured later in life. However, the differences between liberal and radical inequality of opportunity are smaller using PGIs than using measures of observed skills.

**Significance Statement**

We estimate inequality of opportunity according to two key conceptions: liberal inequality of opportunity, which accepts the effects of natural talents on life outcomes as just, and radical inequality of opportunity, which views the effects of natural talents on life outcomes as unjust. Using survey data from the United States and Germany and both polygenic indices (PGIs) and observed skills as proxies for natural talents, we compare their contributions and those of ascribed characteristics to inequalities in education, occupation, and income. We find that radical inequality of opportunity in Germany and the United States is considerably larger than liberal inequality of opportunity. Qualitatively similar results are obtained independent of whether PGIs or observed measures of skills are employed.

## Introduction

Do we live in a just society? Empirical social science research attempts to answer this question through the lens of equality of opportunity, a widely accepted principle of social justice. John Rawls defined “fair equality of opportunity” as the idea that everyone with the same natural talents who is willing to put in the same effort should have the same life outcomes (1). We refer to this conception of (in)equality of opportunity as *liberal inequality of opportunity*. In sociological words, ascribed characteristics such as social origin, migration background, and sex should not affect life outcomes (2). However, in political philosophy, alternative conceptions of inequality of opportunity have been proposed. Luck egalitarian philosophers argue that any inequality resulting from factors beyond the individual’s control is unjust (3–5). Therefore, we refer to *radical inequality of opportunity* as a conception of inequality of opportunity which shares with the principle of liberal inequality of opportunity the idea that it is unjust for ascribed characteristics to affect life outcomes. In addition, radical inequality of opportunity holds that it is unjust for natural talents to affect life outcomes because individuals cannot control them either.

While most empirical social science research has measured liberal inequality of opportunity (6), an approach has been developed to measure radical inequality of opportunity (7). There have also been comparisons between liberal and radical inequality of opportunity (8, 9). A shortcoming of previous work is that it relies on observed skills measured in adulthood to proxy for natural talents. This is problematic because environmental influences and individual choices can affect skills in adulthood, making it difficult to disentangle the contributions of natural talents from those of ascribed characteristics and individual choices. To address this problem, we measure natural talents using polygenic indices (PGIs), since an individual’s DNA sequence is determined at birth and does not change throughout the life course. We compare the use of PGIs to the use of skills measured at a later stage in the life course. In addition, we empirically measure and compare liberal and radical inequality of opportunity in two independent data sources from two different countries (United States [Wisconsin] and Germany).

Our study also contributes to an ongoing debate on the impact of genes on life outcomes. Social science genetics has been dominated by the notion of liberal inequality of opportunity, with some researchers arguing that an increase in the influence of genetic differences on life outcomes implies an increase in equality of opportunity (10, 11). Recently, however, researchers in the field of social science genetics have expressed ideas that are related to the radical notion of inequality of opportunity, arguing that individuals cannot be held responsible for the genetic variants they happen to be born with (12). Furthermore, genetic influences on life outcomes are not fixed but can be affected by policies, further justifying their inclusion into inequality of opportunity (12).

## Methods

### Data

We use survey data from the United States (Wisconsin) and Germany. For the United States, we use the Wisconsin Longitudinal Study (WLS), in which 47.4% of respondents have been genotyped (13). The WLS is a random sample of 10,317 students who graduated from Wisconsin high schools in 1957. The sample for which all relevant information is available consists of N=3,626 individuals. The average respondent in our sample completed 14 years of education. Half of the respondents have an income below 2,500 Dollars/month, 50% of them are female, and 45% have at least one parent who achieved secondary school education. For Germany, we use the Innovation sample from the Socio-Economic Panel Study (SOEP), in which 58% of respondents consented to genotyping (14, 15). The SOEP Innovation sample is a multistage random sample, regionally clustered. 6,576 individuals were invited to participate in the 2019 sample. Among them, 2,598 provided a valid genetic sample, and the number of individuals for which all the relevant information was available was N=776. We removed the biological parents of respondents to avoid excessive genetic similarity (N=725), and we restricted the sample to individuals who were in working age (25–65) at the first outcome measurement, yielding a final sample size of N=589. The average respondent completed 13 years of education. Half of the respondents have an income below 2,400 Euros/month, 53% of them are female, and 66% have at least one parent who achieved secondary school education. Therefore, the two samples are fairly comparable with respect to measures of life outcomes. However, SOEP presents a quite wide age distribution. In the Supplementary material, Table S4 and Fig. S1, we show the results obtained by considering different age ranges separately in SOEP. Results are generally robust across age ranges.

In line with the standard in social science genetics, the analysis in both data sets is limited to respondents with an European genetic ancestry. In both data sets, over 98% of the respondents have a European genetic ancestry, so that this restriction does not affect the generalizability of our results for the populations covered by these data sets.

### Methodology

**Fig. 1.**
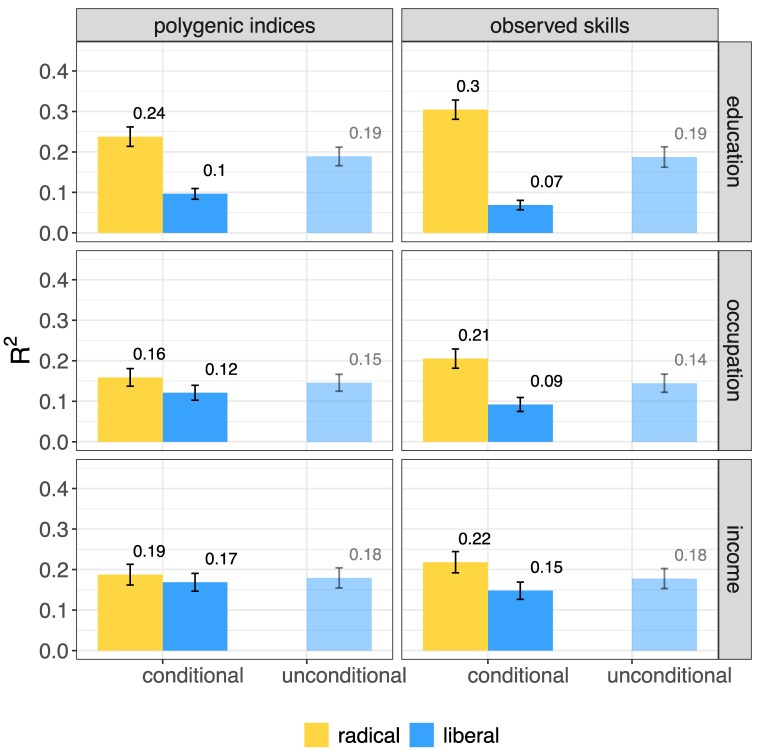
WLS. Explained variance by ascribed characteristics and natural talents (radical inequality of opportunity, yellow), and by ascribed characteristics (liberal inequality of opportunity, blue). The latter is estimated in two different ways, as the *semipartial*  $R^{2}$ conditional on natural talents (“conditional”), and as the total $R^{2}$ of a model containing only ascribed characteristics (“unconditional”). Error bars show bootstrapped 95% CI. N=3,626.

**Fig. 2.**
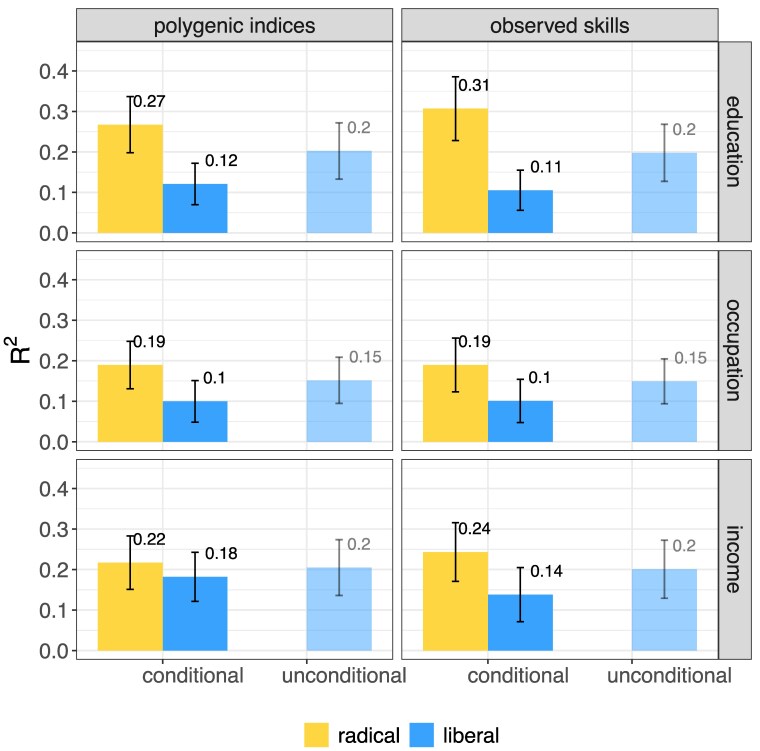
SOEP. Explained variance by ascribed characteristics and natural talents (radical inequality of opportunity, yellow), and by ascribed characteristics (liberal inequality of opportunity, blue). The latter is estimated in two different ways, as the *semipartial*  $R^{2}$ conditional on natural talents (“conditional”), and as the total $R^{2}$ of a model containing only ascribed characteristics (“unconditional”). Error bars show bootstrapped 95% CI. N=589.

We employ a combination of double lasso and bootstrap aggregation (bagging) (16), with multiple linear regressions as the basic model. This approach reduces the risk of overfitting by introducing variability at the observation level through bagging, and at the variable level through the double lasso procedure. All numerical variables are scaled to ensure the comparability of $R^{2}$ across models. For each bootstrap sample, we select variables via double lasso from the complete variables set. We run two multiple linear regression models. The first includes both variables related to ascribed characteristics and to natural talents. The total $R^{2}$ is our measure of radical inequality of opportunity, and the *semipartial*  $R^{2}$ of ascribed characteristics is our measure of (conditional) liberal inequality of opportunity. It is conditional in that it is the unique contribution of ascribed characteristics after controlling for natural talents. To estimate unconditional liberal inequality of opportunity, we run a second model only including the variables selected by double lasso which are related to ascribed characteristics. The total $R^{2}$ of this model is the contribution of ascribed characteristics without controlling for natural talents. Finally, the resulting estimates of inequality of opportunity are the means of the 1,000 bootstrap iterations. Standard errors (in Tables 1 and 2) and 95% CI (in Figs. 1 and 2) are also obtained via bootstrapping. The first ten genetic principal components (17) are included by default in the ascribed characteristics (not part of model selection), as they capture population stratification patterns. The default inclusion of the principal components methodologically justifies the choice of double lasso over simple lasso. While this technique is generally used when there’s a predictor of main interest, which is not subject to model selection, we here use it to perform variable selection while keeping the principal components fixed.

**Table 1.** WLS. Unjust inequalities according to radical and liberal inequality of opportunity.

Natural talents	Outcome	Radical	(SE)	Liberal	(SE)	Difference
Polygenic indices	Education	0.24	(0.01)	0.10	(0.01)	$0.14^{*}$
	Occupation	0.16	(0.01)	0.12	(0.01)	$0.04^{*}$
	Income	0.19	(0.01)	0.17	(0.01)	$0.02^{*}$
Observed skills	Education	0.30	(0.01)	0.07	(0.01)	$0.23^{*}$
	Occupation	0.21	(0.01)	0.09	(0.01)	$0.12^{*}$
	Income	0.22	(0.01)	0.15	(0.01)	$0.07^{*}$

“Liberal” refers to conditional liberal inequality of opportunity. “Difference” is the difference between radical and liberal inequality of opportunity, where * indicates a Holm-corrected *p*-value <0.05 in a paired *t*-test. Estimates of inequality ($R^{2}$) and their standard errors (SE) are obtained by bootstrapping aggregation over 1,000 iterations. N=3,626.

**Table 2.** SOEP. Unjust inequalities according to radical and liberal inequality of opportunity.

Natural talents	Outcome	Radical	(SE)	Liberal	(SE)	Difference
Polygenic indices	Education	0.27	(0.04)	0.12	(0.03)	$0.15^{*}$
	Occupation	0.19	(0.03)	0.1	(0.03)	$0.09^{*}$
	Income	0.22	(0.03)	0.18	(0.03)	$0.04^{*}$
Observed skills	Education	0.31	(0.04)	0.11	(0.03)	$0.20^{*}$
	Occupation	0.19	(0.03)	0.10	(0.03)	$0.09^{*}$
	Income	0.24	(0.04)	0.14	(0.03)	$0.10^{*}$

“Liberal” refers to conditional liberal inequality of opportunity. “Difference” is the difference between radical and liberal inequality of opportunity, where * indicates a Holm-corrected *p*-value <0.05 in a paired *t*-test. Estimates of inequality ($R^{2}$) and their standard errors (SE) are obtained by bootstrapping aggregation over 1,000 iterations. N=589.

### Variables

#### Outcomes

We measure as life outcomes educational attainment (years of education), occupational status (Duncan’s Socio-Economic Index [SEI] score for WLS; International Socio-Economic Index [ISEI] for SOEP), and income (gross monthly wages, salaries, commissions, and tips for WLS; gross monthly labor income for SOEP). For each outcome, we take the maximum value observed for an individual across waves. In WLS, participants were between 34 and 38 years old at the first outcome measurement, while they were between 25 and 65 at the first outcome measurement in SOEP.

#### Ascribed characteristics

For ascribed characteristics, we include the traditional measures in sociology that were available for both datasets: year of birth, sex, migration background, paternal years of education, highest occupational status between both parents. In SOEP, only gender of the respondent is available, instead of biological sex. Additionally, we include the first 10 genetic principal components.

#### Polygenic indices

In WLS, we use all the available PGIs: Educational attainment, cognitive performance, self-reported maths ability, highest-level maths class taken, depression, well being, and neuroticism. In SOEP, we select a comparable set of PGIs: Educational attainment, cognitive performance, highest maths score, self-reported maths score, depression, self-reported health, and neuroticism. Detailed information about GWAS sample size and genotyping procedures for both WLS and SOEP can be found elsewhere (13, 15). We repeated the analyses including all single-trait PGIs available in SOEP to assess the sensitivity of our conclusions to the use of a larger set of PGIs. The results, shown in the Supplementary material, Table S3, show that our conclusions are robust to the inclusion of more PGIs.

#### Observed skills

Cognitive skills in WLS are measured with the percentile rank based on national test takers for the Henmon–Nelson test, which was administered during the junior year of high school, when respondents in WLS were 16–17 years old. In SOEP, we measure cognitive skills as the number of correct numerical entries in 30 seconds in the “Signs and numbers” test. Noncognitive skills include concepts that map onto the ones captured by the PGIs. In WLS, scores for the Big 5 personality traits are reported in wave 4, when respondents were between 52 and 56 years old. In SOEP, we include a set of items for each trait of the Big 5: is original, values artistic experiences, has a lively imagination, is inquisitive (Openness)—thorough worker, tends to be lazy, performs tasks efficiently (Conscientiousness)—communicative, sociable, reserved (Extraversion)—sometimes too rough with others, friendly with others, forgiving (Agreeableness)—worries a lot, somewhat nervous, handles stress well (Neuroticism). Skills in SOEP were measured at different waves, and we take the maximum observed values for each individual. The participants in SOEP were between 17 and 63 when cognitive and noncognitive skills were measured.

The complete list of survey questions for all variables can be found in the *Supplementary material*, Tables S1 and S2.

## Findings

### Radical inequality of opportunity is substantially larger than liberal inequality of opportunity

Figures 1 and 2 show the explanatory power of ascribed characteristics and natural talents in predicting life outcomes (educational attainment, occupational status, and income). It shows how these differ when we measure natural talents using PGIs (left panel) as opposed to using measures of observed skills (right panel). We focus first on the left-hand side of the first panel, which displays radical inequality of opportunity, and liberal inequality of opportunity if natural talents, as measured by PGIs, are controlled for. The exact estimates are reported in Tables 1 and 2.

Ten percent (blue bar) of inequality in education is uniquely due to ascribed characteristics in the WLS and is therefore unjust from the perspective of liberal inequality of opportunity. The corresponding figure in SOEP is 12%.

The additional contribution of natural talents, as measured by PGIs, is about 14% in the WLS and 15% in SOEP. As a result, 24% of educational inequality is unjust from the perspective of radical inequality of opportunity in Wisconsin and 27% in Germany (yellow bar).

Compared to education, occupation, and income show lower levels of radical inequality of opportunity in both the WLS and SOEP. However, liberal inequality of opportunity is higher for income than for any other outcome. The difference between liberal and radical inequality of opportunity is largest for education, followed by occupation (0.04 in WLS, 0.09 in SOEP) and income (0.01 in WLS, 0.04 in SOEP).

Using observed measures of skills leads to very similar conclusions as using PGIs, but with larger gaps between liberal and radical inequality of opportunity. The additional contribution of observed skills to inequality of opportunity is largest for education (0.23 WLS; 0.20 SOEP), followed by occupation (0.12 WLS; 0.09 SOEP) and income (0.07 WLS; 0.10 SOEP). With respect to education, 7% of inequality is related to ascribed characteristics only, and is therefore unjust from the perspective of liberal inequality of opportunity in the WLS, and 11% in the SOEP. From the perspective of radical inequality of opportunity, 30% of inequality is unjust in the WLS, and 31 in the SOEP. Also when we use observed skills instead of PGIs, we find lower levels of radical inequality in occupation and income with respect to education, and slightly higher levels of liberal inequality of opportunity in income with respect to the other outcomes.

### Liberal inequality of opportunity is overestimated by not accounting for natural talents

Research often estimates the effects of ascribed characteristics without controlling for natural talents. Figures 1 and 2 also show how the explanatory power of ascribed characteristics changes when we remove the contribution of natural talents measured by PGIs or observed skills. To do this, we compare two ways of measuring liberal inequality of opportunity: unconditional, which does not control for natural talents, and conditional, which does.

We observe that controlling for PGIs (left panel) always reduces the explanatory power of ascribed characteristics. Thus, not controlling for PGIs overestimates liberal inequality of opportunity. Compared to PGIs, when we control for observed skills we find a similar overestimation. However, the overestimation in liberal inequality of opportunity in income is slightly larger when we measure natural talents using observed skills instead of PGIs. But the substantive conclusions are unaffected by these small differences.

## Discussion

Different conceptions of inequality of opportunity lead to different conclusions about the extent of inequality of opportunity in contemporary societies such as the United States (Wisconsin) and Germany. Radical inequality of opportunity is larger than liberal inequality of opportunity, meaning that natural talents do explain a significant share of differences in life outcomes. This is particularly true for educational attainment. Differences in natural talents are the main driver of educational inequality of opportunity, while ascribed characteristics play a smaller role. The distribution of occupations is generally fairer than that of education, but occupational inequality of opportunity is driven more by ascribed characteristics and less by natural talents, especially in Wisconsin. Income shows slightly higher levels of inequality of opportunity than occupation according to both liberal and radical inequality of opportunity, but most of inequality of opportunity in income is due to ascribed characteristics.

The conclusions are substantively the same whether we measure natural talents using PGIs or using observed skills. We do, however, observe smaller differences between radical and liberal inequality of opportunity when using PGIs. Nevertheless, the explanatory power of PGIs is expected to increase with increasing sample size in GWAS, meaning that the estimates reported here could be lower bound estimates of the contribution of natural talents.

Ignoring the contribution of differences in natural talents to inequality leads to an overestimation of the impact of ascribed characteristics on life outcomes, a crucial matter for research on the intergenerational transmission of advantage. Our measure of conditional liberal inequality of opportunity accounts for the correlation between natural talents and ascribed characteristics, isolating the unique contribution of ascribed characteristics in the explanation of inequalities. The magnitude of the overestimation is slightly smaller using PGIs than using observed skills, which can be partially due to the fact that ascribed characteristics and individual choices can affect observed skills.

Our results highlight the advantages of using genetic data in the study of social inequalities. The intergenerational transmission of advantage occurs through the inheritance of values, financial and social support, but also through the transmission of natural talents (understood in a very general sense as any innate individual characteristic that a society values in terms of education, occupational status, or income) (18). The correlation between parental and offspring PGI can be used to measure the intergenerational transmission of natural talents, since PGIs are not influenced by ascribed characteristics and by individual choices, while achieving a comparable explanatory power as observed skills. Using PGIs improves the estimation of the impact of natural talents on life outcomes, leading to less biased estimates of the relative importance of differences in ascribed characteristics and natural talents in explaining inequalities. In the absence of genetic data, observed skills can be a valid alternative measure of natural talents. Although observed skills are measured later in the life course, the results obtained using them are similar to those obtained using PGIs.

However, the use of PGIs also has limitations. First, while the inclusion of the first 10 genetic principal components partially accounts for population stratification patterns, residual environmental confounding is likely to be present (19). This could result from effects of parental genetic variants, which are not mediated by children’s genetic variants but by parental environmental characteristics, on children’s outcomes, and from assortative mating. For these reasons, the associations between PGIs and life outcomes cannot be interpreted causally (the same applies for the associations between ascribed characteristics and life outcomes as well as the associations between skills and life outcomes). There are two possible ways to address this issue in future work. One way is to control for environmental confounding by using sibling fixed-effects models. The second way is to condition on parental PGIs whilst estimating the effects of own PGIs. Both approaches require data on PGIs within families, which is increasingly available. However, for our data the sample sizes are small (for instance, there are only 152 trios of mother–father–child available in the SOEP data). In addition, these approaches cannot be easily combined with our descriptive methodological approach, which aims to explain the variance of education, occupation, and income using measure of ascribed characteristics and natural talents. But future work may employ these approaches to better identify direct genetic effects (19).

Another issue is that PGIs capture only part of the total association between genetic variants and phenotypes. For this reason, estimates of radical inequality of opportunity could even be lower bound estimates. Concerning observed skills, due to data availability our measures are captured late in life for some individuals, meaning that they are more likely to be affected by ascribed characteristics and individual choices. We believe that any confounding is likely to be bigger for the models employing skills than for the models employing PGIs.

Finally, the methodology we employ requires a priori selection of the variables to be included. With respect to natural talents, we show in the Supplementary material, Table S1, that including a significantly larger number of PGIs in the variable selection routine does not affect our results. We cannot rule out that a more comprehensive set of variables related to ascribed characteristics would affect inequality estimates. However, under the assumption that there are some underlying factors which matter to explain inequalities and are generalizable, the double lasso procedure partially accounts for this eventuality. In fact, its role is to select the main predictors for a given outcome even starting from a larger input set of variables, as shown from the PGIs case.

Even taking into account these limitations, in particular that our results should be interpreted descriptively rather than causally, our findings demonstrate that distinguishing between different conceptions of inequality of opportunity is not only theoretically important but also empirically relevant. The results show that natural talents, alongside factors like financial and social capital, play a meaningful role in leading to inequality in life outcomes. From the perspective of radical inequality of opportunity, both natural talents and ascribed characteristics represent morally arbitrary factors that create unjust inequalities in life outcomes. This implies that current policies focused solely on addressing environmental disadvantages may be insufficient for achieving equality of opportunity.

In addition, the associations we observe between genetic variants and life outcomes are not fixed but can be changed through interventions. Just as social policies can modify the impact of family background on life outcomes, they can also modify how genetic variants translate into outcomes (20). As genetic data will be increasingly used together with social science data, society will need to grapple with these philosophical questions about inequality of opportunity in ways that recognize both the contribution of genetic variants to inequality of opportunity, as well as their malleability through environmental interventions.

## Supplementary Material

pgaf140_Supplementary_Data

## Data Availability

The data are available via DIW Berlin (https://www.diw.de/en/diw_01.c.601584.en/data_access.html) and the WLS website(https://researchers.wls.wisc.edu/data/survey-data/wisconsin-longitudinal-study-data-access/). The R-scripts to replicate all analyses are available at https://osf.io/526kq/.

## Previous Presentation

These results were previously presented at workshops in Venice, Oslo, and Rotterdam, at the RC28 conference in Providence, RI, and at the LIVES conference in Geneva in 2024.

## Preprints

A preprint of this article is published at https://osf.io/preprints/socarxiv/2t5zu_v3.
