# Supplementary material for: The role of polygenic indices in inequality of opportunity
Source: PNAS Nexus. 2025 May 5;4(5):pgaf140. doi: 10.1093/pnasnexus/pgaf140 (PMC12076009; doi:10.1093/pnasnexus/pgaf140)
Supplement: pgaf140_Supplementary_Data [file pgaf140_supplementary_data.pdf]

Supplementary Material for **The Role of Polygenic Indices in Inequality of Opportunity, published in PNAS Nexus**

Michael Grätz and Sonia Petrini, University of Lausanne and Stockholm University

Survey Questions

In Tables S1 and S2 we report the full description of the original survey questions related to our variables of interest. Refer to the main text for details on how the variables have been aggregated, and for the list of polygenic indices.

Table S1: WLS. Survey questions. "R03" indicates, for instance, "Round 3". "R" stands for"respondent","SD" stands for "Status and Descriptive Variables"

**Category**	**Variable**	**Label**
Outcomes	Education	R03 Equivalent years of regular education<br>R04 Summary of equivalent yrs of regular education based on most recent degree<br>R05 How many years of education does R have based on his or her highest degree?<br>R06 Summary of equivalent years of regular education based on highest degree
	Income	R04 Past yr, how much wages received before taxes?<br>R05 R wage amount in the last 12 months<br>R06 R wage amount in the last 12 months
	Occupation	R02 1950 Duncan SEI score for graduate's occupation<br>R03 1970 Duncan SEI score for current or last job<br>R04 1970 Duncan SEI score for current/last job
Ascribed characteristics	Year of birth	SD Year of participants' birth
	Sex	SD Sex of participant
	Migration background	R05 Were you born in Wisconsin?
	Paternal years of education	R01 Father's years of schooling
	Parental occupational status	R03 1970 Duncan SEI score for mother's job in 1957<br>R03 Preferred measure for father's Duncan SEI score for 1957 job
Observed Skills	Cognitive	Percentile rank based on national test takers for the Henmon-Nelson test
	Non-cognitive	R04 Summary score for Extraversion<br>R04 Summary score for Openness<br>R04 Summary score for Neuroticism<br>R04 Summary score for Conscientiousness<br>R04 Summary score for Agreeableness

Table S2: SOEP. Survey questions.

**Category**	**Variable**	**Label**
**Outcomes**	Education	Amount of education or training (in years)
	Income	Current gross labor income in euros
	Occupation	ISEI-Status following Ganzeboom (based on IS88)
**Ascribed characteristics**	Year of birth	Birth Year, 4-digit
	Sex	Gender
	Migration background	Born in Germany
	Paternal years of education	Level Of Education Father
	Parental occupational status	MOTHER: ISEI-Status following Ganzeboom (based on IS88)<br>FATHER: ISEI-Status following Ganzeboom (based on IS88)
**Observed Skills**	Cognitive	Number of correct numerical entries in 30 seconds in the "Signs and numbers" test
	Non-cognitive	Extraversion:<br>- communicative<br>- sociable<br>- reserved<br>Openness:<br>- is original<br>- values artistic experiences<br>- has a lively imagination<br>- is inquisitive<br>Neuroticism:<br>- worries a lot<br>- somewhat nervous<br>- handles stress well<br>Conscientiousness:<br>- thorough worker<br>- tends to be lazy<br>- performs tasks efficiently<br>Agreeableness:<br>- sometimes too rough with others<br>- friendly with others<br>- forgiving

Sensitivity Analyses

Increasing the number of polygenic indices

While in our conceptual framework we focus on the idea of “natural talents” as mainly related to cognitive and non-cognitive skills, genetic differences related to health and psychiatric conditions also affect life outcomes and are beyond an individual’s control. In a robustness check, we exploit the large number of polygenic indices available in SOEP to assess the sensitivity of our inequality of opportunity estimates to the inclusion of more PGIs. Table S3 shows the inequality estimates obtained when all the 55 single-trait PGIs available in SOEP are included in the model selection procedure at each bootstrap iteration. The complete list of single-trait PGIs can be found elsewhere (Koellinger et al., 2023). For all the outcomes, the additional PGIs increase estimates of radical inequality of opportunity by 2-3 percentage points. However, both the estimates of radical and liberal inequality of opportunity fall within the 95% bootstrapped confidence intervals reported in the main analyses, where only 7 PGIs are included. This suggests that the majority of the variance is already captured by the reduced set of PGIs. Thus, adding more PGIs does not substantially change the estimates of inequality of opportunity.

Table S3: Unjust inequalities according to radical and liberal inequality of opportunity in SOEP, including all the available PGIs in the variable selection process. “Liberal” refers to conditional liberal inequality of opportunity. “difference” is the difference between radical and liberal inequality of opportunity, where * indicates a Holm-corrected p-value < 0.05 in a paired t-test. Estimates of inequality (R^2) and their standard errors (SE) are obtained by bootstrapping aggregation over 1,000 iterations. $N = 589$.

Natural talents	Outcome	Radical	(SE)	Liberal	(SE)	difference
Polygenic indices	education	0.29	(0.04)	0.11	(0.03)	0.18*
	occupation	0.22	(0.03)	0.10	(0.03)	0.12*
	income	0.24	(0.04)	0.16	(0.03)	0.08*

Stratifying by age

Our estimates of inequality of opportunity could differ by the age of the respondent. We exploit the variability in the SOEP data to investigate this question. Hence, we repeat the analyses only focusing on the bottom half of the age distribution, that is with age below median (from 25 to 45 years old), and on the top half of the distribution (from 46 to 65 years old). Table S4 shows the resulting estimates for radical and (conditional) liberal inequality of opportunity, while Figure S1 compares these estimates to those obtained with the full age range. The resulting estimates are consistently within the 95% bootstrapped confidence intervals reported in the main analyses, with the exception of radical inequality of opportunity in income, which is 29 percent in the young age range, while it is 17 percent for the older age range.

Table S4: Unjust inequalities according to radical and liberal inequality of opportunity in SOEP, stratified by age. “Liberal” refers to conditional liberal inequality of opportunity. “difference” is the difference between radical and liberal inequality of opportunity, where * indicates a Holm-corrected p-value < 0.05 in a paired t-test. Estimates of inequality (R^2) and their standard errors (SE) are obtained by bootstrapping aggregation over 1,000 iterations. $N = 589$.

Age	Natural talents	Outcome	Radical	(SE)	Liberal	(SE)	difference
25 - 45	Polygenic indices	education	0.26	(0.05)	0.16	(0.05)	0.1*
		occupation	0.23	(0.05)	0.15	(0.04)	0.08*
		income	0.29	(0.05)	0.22	(0.04)	0.07*
46 - 65	Polygenic indices	education	0.30	(0.05)	0.14	(0.04)	0.16*
		occupation	0.20	(0.04)	0.13	(0.04)	0.07*
		income	0.17	(0.05)	0.15	(0.05)	0.02*

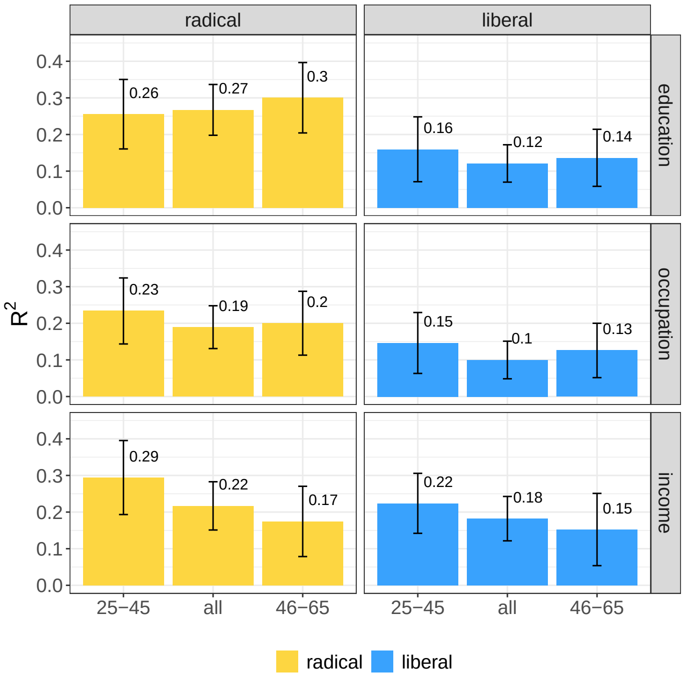

Figure S1: SOEP. Explained variance by ascribed characteristics and natural talents (radical inequality of opportunity, yellow), and by ascribed characteristics (liberal inequality of opportunity, blue), for different age spans. Natural talents are measured via polygenic indices. "all" refers to the full age range from 25 to 65 years old, as used in the main analyses. Error bars show bootstrapped 95% confidence intervals. $N = 589$.

References

Koellinger, P. D., Okbay, A., Kweon, H., Schweinert, A., Linnér, R. K., Goebel, J., Richte, D., Reiber, L., Zweck, B. M., Belsky, D. W., et al. (2023). Cohort profile: Genetic data in the german socio-economic panel innovation sample (soep-g). *Plos one*, 18(11):e0294896.
